# Theta Oscillations Alternate With High Amplitude Neocortical Population Within Synchronized States

**DOI:** 10.3389/fnins.2019.00316

**Published:** 2019-04-12

**Authors:** Erin Munro Krull, Shuzo Sakata, Taro Toyoizumi

**Affiliations:** ^1^RIKEN Center for Brain Science, Tokyo, Japan; ^2^Beloit College, Beloit, WI, United States; ^3^Strathclyde Institute of Pharmacy and Biomedical Sciences, University of Strathclyde, Glasgow, United Kingdom

**Keywords:** synchronized state, independent component analysis, theta oscillation, non-REM, slow oscillation, delta oscillation, cortical state

## Abstract

Synchronized states are marked by large-amplitude low-frequency oscillations in the cortex. These states can be seen during quiet waking or slow-wave sleep. Within synchronized states, previous studies have noted a plethora of different types of activity, including delta oscillations (0.5–4 Hz) and slow oscillations (<1 Hz) in the neocortex and large- and small- irregular activity in the hippocampus. However, it is not still fully characterized how neural populations contribute to the synchronized state. Here we apply independent component analysis to parse which populations are involved in different kinds of neocortical activity, and find two populations that alternate throughout synchronized states. One population broadly affects neocortical deep layers, and is associated with larger amplitude slower neocortical oscillations. The other population exhibits theta-frequency oscillations that are not easily observed in raw field potential recordings. These theta oscillations apparently come from below the neocortex, suggesting hippocampal origin, and are associated with smaller amplitude faster neocortical oscillations. Relative involvement of these two alternating populations may indicate different modes of operation within synchronized states.

## Introduction

Cortical state can be described along a spectrum from synchronized to desynchronized (Harris and Thiele, 2011). Desynchronized states are characterized by low amplitude high frequency activity where local neuronal activity is uncorrelated. Desynchronized activity is seen during active waking (engaging in a task such as navigating a maze) and rapid-eye movement sleep (REM). In rats, while the neocortex is desynchronized, the hippocampus exhibits theta oscillations, sinusoidal oscillations between 4 and 8 Hz (Diekelmann and Born, 2010). Synchronized states are characterized by high-amplitude, low-frequency oscillations where neuronal populations fluctuate between UP states marked by frequent neuronal firing, and DOWN states marked by neuronal silence. Synchronized states are seen during quiet waking (resting or engaging in a routine task such as eating) as well as non-REM sleep. In rats, while the cortex is synchronized, the hippocampus exhibits irregular activity featuring sharp waves, quickly depolarizing waves generated in CA3 accompanied by ripple oscillations (>100 Hz) (Diekelmann and Born, 2010). Within non-REM sleep, neocortical activity progresses from K-complexes (large amplitude biphasic waves) to slow-wave activity (SWA) (<1 Hz) mixed with delta oscillations (1–4 Hz) (Crunelli et al., 2006). At the same time, the hippocampus can display either low- or high-amplitude irregular activity (Miyawaki et al., 2017). While activity during synchronized states has been widely studied, neural ensemble dynamics within synchronized states are not still fully characterized (Diekelmann and Born, 2010; de Andrés et al., 2011; Crunelli et al., 2015; Neske, 2016; Mizuseki and Miyawaki, 2017).

**FIGURE 1:**
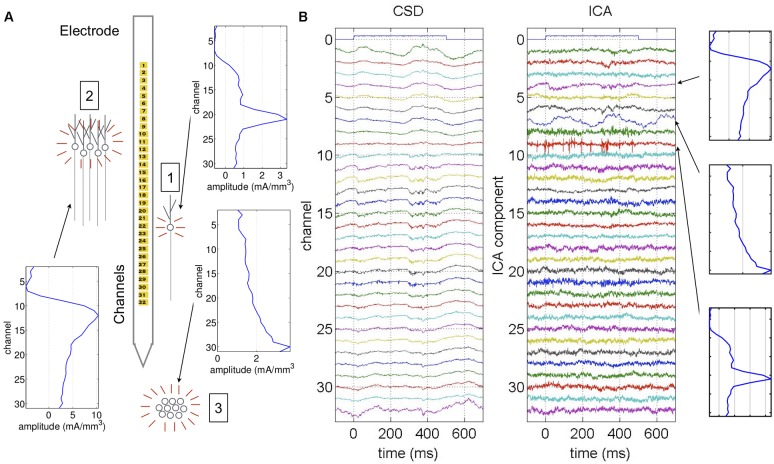
Example of ICA applied to neural data. Data generated from a single anesthetized experiment used as an example throughout this study. **(A)** Different populations generate field potentials across the electrode, resulting in overlapping signals recorded at each channel. For example, the signal from a single cell (1) may have the highest amplitude in a single channel, while other channels have much lower amplitude. The signal from a larger population (2) may have high amplitude across multiple channels with a single dipole. A population below the electrode (3) may generate the highest amplitude signal in the channel closest to the population, which decays with channels further from the population and doesn’t display a dipole (Herreras et al., 2015). **(B)** Example CSD from 32-channel electrode along with ICA decomposition. The ICA components are listed in order from largest to smallest amplitude over the entire experiment. In the CSD, the auxiliary channel 0 indicates a tone is played at time 0, and we see an immediate reaction to the stimulus in channels 11 through 17. This immediate reaction to the tone is summarized in ICA component 4. Each ICA component is associated with an anatomical map showing the amplitude of that component across the electrode. The anatomical map for ICA component 4 is consistent with the large population (2) in **(A)**. Similarly, high frequency activity seen in the CSD in channels 20 and 21 is reflected in ICA component 9, whose anatomical map is consistent with the single cell (1) in **(A)**. Finally, there is a 4 Hz sinusoidal oscillation in ICA component 7 that is not apparent in the original CSD. The anatomical map gradually increases toward the lowest channel and there is no dipole, indicating that the oscillation was generated from below the electrode as in population (3) in **(A)**.

Previous studies on cortical dynamics primarily analyzed activity by looking at either spiking activity or the local field potential (LFP) (Einevoll et al., 2013). Single- or multi-unit spiking activity gives detailed information about individual neural interactions. However, because the number of neurons we can detect is limited, we must infer how cell populations behave as a whole. On the other hand, LFP recordings reflect activity from many populations simultaneously, including neurons and non-spiking glia (Buzsáki et al., 2012). However, signals from different populations tend to overlap at each recording site, as illustrated in Figure 1. The fact that signals overlap makes these recordings difficult to parse, i.e., it isn’t clear which populations are involved in different kinds of activity (Kajikawa and Schroeder, 2011; Buzsáki et al., 2012; Kajikawa and Schroeder, 2015). Current source density (CSD) analysis helps reduce signal spread by focusing on current sinks and sources, but it may still be difficult to parse which cell populations are involved, particularly in the neocortex where neuronal populations are very dense (Gulyás et al., 2016).

To address the issue of overlapping population signals, we turned to independent component analysis (ICA) (Brown et al., 2001). Based on the assumption that the LFP is made up of summed population signals, and that the relative amplitude of a single population across the electrode remains fixed over time, ICA decomposes recordings so that signals from different populations are separated into different components. ICA works by finding a matrix that decouples the signals so that they are as statistically independent as possible. Since ICA decouples signals by matrix multiplication, the number of extracted components is the same as the number of recording channels. If there are more populations than recording channels, then ICA groups populations with similar spatial profiles together in the same component. Even with a limited number of channels, ICA has proven to be very effective for parsing signals from different types of neural recordings (Jung et al., 2001; Makarov et al., 2010; Herreras et al., 2015). Moreover, unlike principal component analysis which focuses on high amplitude signals, ICA can easily parse low amplitude signals that may be missed from the raw LFP.

In this study, we applied ICA to neocortical laminar recordings from unanesthetized and urethane anesthetized rats, and consistently observed two ICA components that alternate throughout synchronized states. One component has a high amplitude signal that broadly covers much of the lower layers (broad layer 5, BL5); the other component shows intermittent theta oscillations and has an anatomical profile indicating it is below the neocortex (THETA) (Herreras et al., 2015). Furthermore, we found that during synchronized states when BL5 is active neocortical UP/DOWN states tend to be slower and have a larger amplitude, while faster small-amplitude UP/DOWN states are seen when SUB is active. These results suggest two modes of operation within the brain during cortical synchronized states.

## Materials and Methods

### Animals

We used twenty-three adult Sprague-Dawley rats (male, 270–413 g, *n* = 18 for anesthetized experiments, *n* = 5 for unanesthetized experiments). All procedures for anesthetized experiments were performed in accordance with the UK Animals (Scientific Procedures) Act of 1986 Home Office regulations and approved by the Home Office (PPL60/4217 and 70/8883). All procedures for unanesthetized experiments (Sakata and Harris, 2009, 2012) were approved by the Institutional Animal Care and Use Committee of Rutgers University.

### Electrophysiological Experiments

Detailed experimental procedures are described in previous studies (Sakata and Harris, 2009, 2012; Sakata, 2016). Briefly, for anesthetized experiments, we anesthetized animals with 1.5–1.6 g/kg urethane. We also administered lidocaine (2%, 0.1–0.3 mg) subcutaneously at the site of incision. After attaching a head-post in the frontal region with bone screws, one of which was used as an electrode for cortical electroencephalograms (EEGs), we placed the animal in a custom head restraint that left the ears free and clear. Two additional bone screws were implanted in the cerebellum as ground. Body temperature was maintained at 37°C with a feedback temperature controller (40-90-8C, FHC). After reflecting the left temporalis muscle, we removed the bone over the left auditory cortex (AC) and carefully performed a small duratomy for each site.

For unanesthetized experiments, in initial surgery, we anesthetized animals with ketamine (100 mg/kg) and xylazine (10 mg/kg), and placed them in a stereotaxic apparatus (David Kopf Instruments). We attached a head-post (Thorlabs, Inc.) with dental cement (3M ESPE, RelyX Luting Cement), removed the left temporal muscle, and covered the exposed bone over the left AC with biocompatible glue and dental cement. After a recovery period, animals were lightly water-deprived, and handling (5–10 min/day) and head-fixation training began. We trained the rats for at least five sessions, during which the duration of restraint was gradually extended. Ten percent sucrose was frequently given during training and water was freely available for at least 1 h after daily training. On the day of recording, we carefully performed a craniotomy and duratomy under isoflurane anesthesia (5% for induction and 0.8% for maintenance). Neither skin nor muscle was cut during this surgery. After a short recovery period (>1 h), recording began.

During recording for both experiments, we covered the brain with 1% agar/0.1 M phosphate buffered saline (PBS) to keep the cortical surface moist. We inserted a 32 channel silicon probe (A1x32-10mm-50-177-A32, NeuroNexus Technologies) where channels are 50 μm apart, either manually or slowly (2 μm/sec or slower) with a motorized manipulator (DMA-1511, Narishige) into the AC (1400–1770 μm from the surface). All electrophysiological experiments were performed in a single-walled soundproof box (MAC-3, IAC Acoustics) with the interior covered by 3 inches of acoustic absorption foam. Broadband signals (0.07–8 kHz) from the silicon probes were amplified (1000 times) (Plexon, HST/32V-G20 and PBX3), digitized at 20 kHz and stored for offline analysis (PXI, National Instruments).

A typical recording schedule was as follows: after insertion of the probe and an additional waiting period (at least 30 min), we started recording with a silent period (at least 5 min), followed by sound presentations, and ended with another silent period (at least 5 min).

Acoustic stimuli were generated digitally (sampling rate 97.7 kHz, TDT3, Tucker-Davis Technologies) and delivered in free-field through a calibrated electrostatic loudspeaker (ES1) located approximately 10 cm in front of the animal. We calibrated the tone presentations using a pressure microphone (PS9200KIT-1/4, ACO Pacific, Inc.) close to the animal’s right ear. Acoustic stimuli for this study consisted of short pure tones (50 ms long with 5 ms cosine ramps, 1/6 or 1/8 octave steps, 3–48 kHz, 10 dB steps, 0–80 dB SPL), long pure tones (300 ms with 10 ms cosine ramps), and unanesthetized experiments also contained brief click trains.

### Histology

After electrophysiological experiments, we perfused rats transcardially with physiological saline followed by 4% paraformaldehyde/0.1 M phosphate buffer, pH 7.4. After overnight post-fixation in the same fixative, we incubated brains in 30% sucrose solution for cryoprotection, cut into 100 μm coronal sections with a sliding microtome (SM2010R, Leica), and the sections were collected and placed in 0.1 M PBS. For verification of silicon probe placement, the free-floating sections were counterstained with NeuroTrace (1/500, N-21480, Life Technologies) in PBS with 0.1% Triton X-100 for 20 min at room temperature. The sections were mounted on gelatin-coated slides and cover-slipped with antifade solutions.

### Data Analysis Preprocessing

We performed all analysis using Matlab (R2017a, Mathworks, Waltham, MA, United States). Since the sample rate of the original recordings was 20 kHz, we first filtered the data below 650 Hz using the fast Fourier transform (FFT, fft in Matlab) with a bump function pass band, where the bump function smoothly transitioned from 0 to 1 over 0.25 Hz. We then removed 50 Hz line noise by linearly interpolating the Fourier transform. Finally, we down-sampled the data to 2 kHz, and high-pass filtered the data over 0.1 Hz using FFT with a bump function pass band.

We noted three kinds of artifacts to remove from analysis: line drift, electrode pops, and epochs with low firing rates. To mark epochs with line drift (large amplitude deviations in the LFP for a single channel), we calculated the standard deviation of each channel. We then marked sections that exceeded 5 standard deviations for that channel. For electrode pops (large jumps in the LFP), we calculated histograms of voltage slopes over the entire experiment for each channel. We marked points where the slope was >2,500 V/s for at least one channel, or >1,000 V/s for at least 2/3 of the channels within 1 ms. To determine overall firing rate, we summed the multi-unit activity (MUA) over all channels, and then computed the average firing rate of the summed MUA over 6 s windows shifted by 600 ms. We marked points that were below 40 Hz for anesthetized experiments and 100 Hz for unanesthetized experiments, where these thresholds were set by the distribution of firing rates for anesthetized and unanesthetized experiments, respectively.

### UP State Detection

We used MUA to determine UP and DOWN states. First we calculated the smoothed MUA (sMUA) by summing the MUA over all channels. We then smoothed the summed MUA using a Gaussian filter with standard deviation of 6.25 ms for anesthetized data and 2.5 ms for unanesthetized data (0.25 divided by minimum firing rate). We then computed UP and DOWN state transitions based on Sakata and Harris (2009). The threshold for transitioning from a DOWN to an UP state was the geometric mean of the sMUA over points where sMUA > 0, while the threshold for transitioning from an UP to a DOWN state was 1/5 the UP state threshold. The minimum UP/DOWN state length was 50 ms for anesthetized data and 25 ms for unanesthetized data.

### Spectrogram Calculation

All spectrograms were calculated over 6 s windows shifted by 600 ms using Welch’s power spectral density estimate (pwelch in Matlab). Spectrograms were used to analyze frequency content of the LFP as well as ICA components. To estimate synchronized states, we computed the spectrogram of the LFP for each channel and then took the median over all channel spectrograms, keeping time and frequency fixed. The low-frequency LFP power (LFP-Power) is the summed power from 1 to 5 Hz (Harris and Thiele, 2011) of the median spectrogram. The threshold for synchronized states for all experiments was set to the median LFP-Power over unanesthetized experiments.

### ICA Application

We first spatially filtered the data using a Gaussian kernel with width 50 μm. We then calculated the spline inverse CSD over the LFP (Pettersen et al., 2006). Since the CSD partially separates sources, previous work suggests that ICA separates the CSD more cleanly than the LFP (Lȩski et al., 2010). For the ICA training data, we randomly selected 100s worth of data points from synchronized data only. We used the extended infomax algorithm to minimize effects of kurtosis in the data (Lee et al., 1999) implemented in EEGLAB (Schwartz Center for Computational Neuroscience, La Jolla, CA, United States). We applied ICA 10 times, each on different training data, to yield 10 different sets of ICA components. We then chose the ICA component set that had minimal mutual information between components (Kraskov et al., 2004). To estimate mutual information, we took samples of 100 to 1,000 s of data points. We then calculated a mutual information factor (MIF) by estimating entropy of individual components, using bins with approximately 10 data points per bin. The MIF is the summed entropy of all individual components, minus ln| W| where W is the ICA unmixing matrix.

Out of the 32 components generated by ICA, we selected the THETA component first and then the BL5 component. ICA lists components according to amplitude. We chose the first component with a narrow band oscillation between 2–5 Hz in anesthetized experiments and 5–9 Hz in unanesthetized experiments, reflecting theta ranges in urethane anesthetized and unanesthetized conditions. The BL5 component is the highest amplitude component, not including the SUB component.

Independent component analysis component amplitude can vary based on experiment as well as populations included in the component. Therefore, we scaled BL5 and SUB according to their activity levels. We considered BL5 active when its signal was correlated with the sMUA. We calculated the correlation over 6 s windows shifted by 600 ms to match spectrogram data. Since the ICA algorithm guesses the sign of a component based on the spatial profile, we switched the sign of BL5 if necessary so that the correlation was negative overall. Time points with correlation ≥0 were considered inactive. If there were fewer than 30 points that met this criterion, then points with correlation ≥-0.1 were considered inactive. We then centered the signal based on the median and interquartile range of inactive points. To measure the activity level of the THETA component, we first determined the peak frequency between 2–5 Hz for anesthetized data and 5–9 Hz for unanesthetized data. We then smoothed the peak frequencies using a Gaussian with standard deviation 10 s, weighting each point according to the amplitude of the peak. We then found the total amplitude around the peak (width 1 Hz for anesthetized, 2 Hz for unanesthetized), along with the amplitude of the surrounding frequencies which consisted of a half width window above and below the peak window. The activity level of THETA was defined as the normalized peak amplitude, where amplitude was centered on the median and interquartile range of the surrounding frequencies over the entire experiment for anesthetized data. Because surrounding frequency amplitude varied a great deal over the course of unanesthetized experiments, we decided to normalize the peak amplitude based and median and interquartile range over 6s instead of the whole experiment.

### Sleep Parameter Threshold Estimation

We estimated sleep stage parameters on the LFP median spectrogram for unanesthetized experiments. We first calculated the LFP delta (0.5–4 Hz), theta (6–9 Hz), and sigma (10–14 Hz) power (Benington et al., 1994; Gross et al., 2009). We then smoothed the power using a Gaussian kernel with width 2.5 s. As sigma × theta power indexes waking vs. sleeping, we selected the sigma × theta threshold first based on largest population density (Benington et al., 1994). We calculated a histogram with 100 bins over the lowest 90% of data points. Then we smoothed the histogram using a Gaussian with width over 1 bin. We calculated the max height of the histogram h_c_ and mean height of the histogram h_m_. The threshold is the first point above h_c_ where the histogram falls below (h_c_+h_m_)/2 as sigma × theta increases. The delta/theta threshold, which indicates the border between REM and non-REM sleep, was based on the non-REM to REM indicator value (NIV) (Benington et al., 1994). We calculated candidate transition to REM (NRT) segments where delta power dropped [delta-DP < 1 in Benington et al. (1994)] and sigma × theta is above threshold. For each NRT segment, we calculated the NIV and the minimum delta/theta value over the entire segment (DT-min). The threshold was set to the median DT-min over all segments with NIV values in the top 50%.

### Laminar Estimation

To display channel data, we estimated the central L4 channel based on the maximum CSD amplitude evoked by the preferred tones. The channel with the highest amplitude was labeled as a thalamic recipient layer (L4).

### Statistical Analysis for Neocortical Activity

To compare neocortical activity across states, we used 2-way ANOVAs based on conglomerated artifact-free data points across all anesthetized or unanesthetized experiments with BL5/THETA index φ between -45 and 135 degrees. All 2-way ANOVAs were performed on the variable of interest as a function of LFP-Power and φ, where LFP-Power was considered continuous and φ was split into BL5 (φ ≤ 45) and THETA (φ > 45). For UP state location, we also included the experiment as a random factor.

UP phase amplitude was defined as the largest amplitude occurrence after Gaussian smoothing over time with a kernel of 2 ms. Peak frequency was defined as the maximum frequency after Gaussian smoothing with a kernel of 0.25 Hz. Peak frequency width was determined by the Matlab function findpeaks.

## Results

We used ICA to investigate which cell populations are active during synchronized states in 18 urethane anesthetized and 5 unanesthetized head-fixed rats. Anesthetized rats exhibited sleep-like states (Clement et al., 2008; Pagliardini et al., 2013), while unanesthetized rats could be waking or sleeping. We recorded from all layers of the AC using 32-channel linear probes (Sakata and Harris, 2009, 2012). From these recordings, we calculated the CSD before applying ICA in order to get clearer components (Lȩski et al., 2010). We applied ICA to the CSD 10 times, each time with different randomly selected sets of artifact-free training data, and chose the ICA run with the least estimated mutual information between components (Kraskov et al., 2004) to promote as much separation as possible.

### Two Populations Consistently Revealed by ICA

We consistently noticed two ICA components. One component exhibited sinusoidal oscillations in the theta range (Buzsáki, 2002; Buzsáki and Draguhn, 2004; Lakatos et al., 2005; Montgomery et al., 2009) that appeared to originate below the neocortex (THETA). The other was the highest amplitude component which broadly affected all layers, centered on layer 5 (BL5).

**FIGURE 2:**
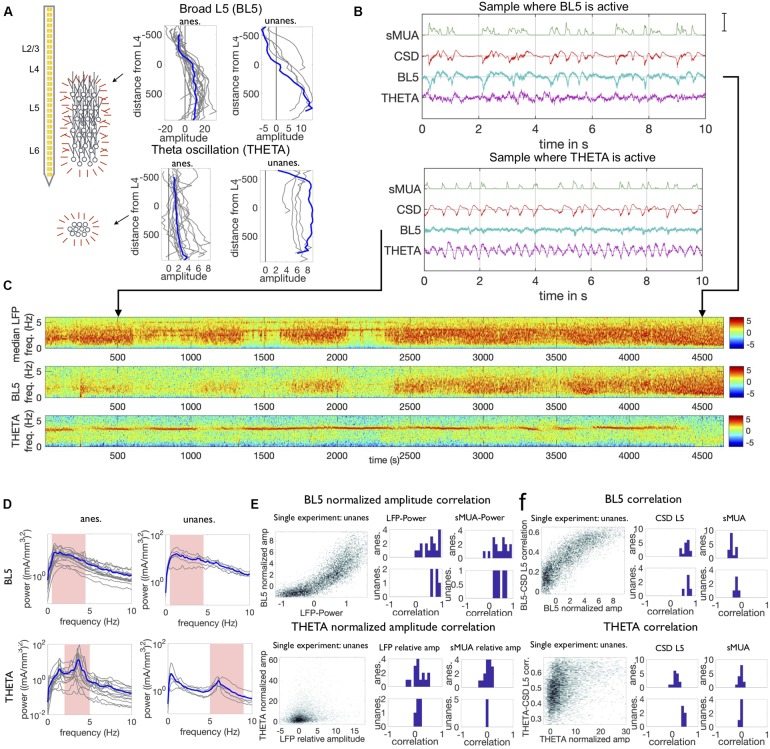
Properties of BL5 and THETA components. **(A)** Spatial maps of both components across 32 channels used to record the LFP, along with diagram of putative population sources. Results for the example anesthetized experiment shown in **(C)** and unanesthetized experiment in Supplementary Figure 2 are highlighted in blue throughout this study. Anatomical maps indicate BL5 is made up of a larger population located within the neocortex, while THETA may be generated by a population below the neocortex. Amplitude in mA/mm^3^, distance in μm. **(B)** Segments when BL5 is active (strong low-frequency oscillations) and when THETA is active (strong sinusoidal oscillation of approximately 4 Hz). Note that the CSD and smoothed multi-unit spiking activity (sMUA) show low-frequency oscillations in both cases. Scale bar is 85th percentile amplitude of sMUA, CSD in channel 17, and ICA component taken from channel with maximum amplitude in anatomical map. **(C)** Spectrograms of the LFP, BL5, and THETA components for an example anesthetized experiment. **(D)** Average frequency spectra when BL5 and THETA are active (scaled amplitude ≥2). Frequencies used to determine activity level are highlighted in pink. **(E)** Correlation between BL5 normalized amplitude with LFP-Power and sMUA low frequency power (1–5 Hz), and THETA normalized amplitude with LFP and sMUA relative theta amplitude. Density plot for example experiments on the left, histograms of correlation from density data on the right. **(F)** Correlation between BL5/THETA signal and CSD from L5 (channel 350 μm below L4) and sMUA. Example experiments on left, histograms of median correlations over points where BL5/THETA are active on the right.

We define the THETA component as the highest-amplitude component with clock-like sinusoidal oscillations in the theta range (2–5 Hz anesthetized, 5–9 Hz unanesthetized). We found a THETA component in 16/18 anesthetized experiments and 5/5 unanesthetized experiments. Figure 2A shows that the amplitude of the THETA component did not have a clear dipole and gradually increased toward the lowest channel in anesthetized experiments, suggesting that signals volume conducted from below the neocortex (Sirota et al., 2008; Kajikawa and Schroeder, 2011; Herreras et al., 2015). In unanesthetized experiments, the amplitude also did not have a dipole and was even over all channels. These anatomical maps were unique in that they were the only components without a dipole (see Supplementary Figure S1). At the same time, we noticed that signals associated with motion, which affects all channels evenly, were also separated into the THETA component. (Note that motion-associated signals may be present outside time intervals marked as motion artifact, see section “Materials and Methods.”) Therefore, it is possible that ICA grouped theta oscillations and motion signals together because of their similar anatomical maps.

We define the BL5 component as the largest amplitude component, excluding the THETA component. Because BL5 was simply the largest component, each experiment had a BL5 component. Anatomical maps of BL5 consistently had large amplitude across all lower layers, with the largest amplitude in L5 and a dipole in L4. These anatomical maps are consistent with a large population generating the signal across multiple channels as illustrated in Figure 2A. BL5 and THETA were active intermittently, as shown in the example anesthetized experiment in Figure 2B,C. When BL5 was active, it exhibited large amplitude low frequency oscillations that closely resembled the CSD. Because of this, we define the BL5 amplitude to be the amplitude from 0.5–4.5 Hz (anesthetized) or 0.5–7 Hz (unanesthetized). When THETA was active, it exhibited a sharp peak frequency between 2–5 Hz (anesthetized) or 5–9 Hz (unanesthetized). We therefore define the THETA amplitude based on the peak amplitude within the theta range (see section “Materials and Methods”). Figure 2D shows the average frequency spectra of both BL5 and THETA when they are active, as defined below.

In order to compare activity level across experiments, we normalized the BL5 and THETA amplitude according to the noise level inherent in their signals. We found that BL5 has a high correlation with the CSD taken from the center of L5 and smoothed Multi-Unit Activity (sMUA) when active, and was uncorrelated with CSD and sMUA otherwise. Therefore, we found the median and inter-quartile range of the BL5 amplitude over all time points where BL5 had low correlation with the sMUA. We then normalized the BL5 component using this median and inter-quartile range (see section “Materials and Methods”). Likewise, in the THETA spectrogram in anesthetized experiments, there was a sharp theta peak which stood out among other frequencies when THETA was active, and blended in with surrounding frequencies when it was not. We therefore found the median and interquartile range of the summed amplitude from frequencies above and below the theta range over the entire experiment. We then normalized the THETA amplitude using the surrounding frequency amplitude median and inter-quartile range (see section “Materials and Methods”). In unanesthetized experiments, while the theta peak stood out during desynchronized states and intermittently during synchronized states, the surrounding frequencies changed according to overall low-frequency LFP power (LFP-Power, 1–5 Hz power of median spectrogram over all channels) (see Supplementary Figure 2). In order to not overestimate the THETA amplitude during synchronized states, we normalized THETA amplitude according to surrounding frequencies within a 6 s window. Since both BL5 and THETA are normalized according to the median and interquartile range of their inherent noise level, we consider BL5 and THETA to be active if their amplitude is ≥2.

Since anatomical maps indicate that BL5 has neocortical origin while THETA originates from below the neocortex, we investigated the relationship between BL5 and THETA and the local activity. In order to investigate the relationship, we looked at both the correlation between the amplitudes of BL5 and THETA with LFP-Power as well as the direct correlation between the component signals and CSD and sMUA. We did this because, while the amplitude focuses on a relevant frequency range, the raw signals may also reveal correlations. We found that the BL5 scaled amplitude is correlated with LFP-Power (*T*-TEST, ANESTHETIZED: *N* = 17, *P* < 0.0001, UNANESTHETIZED: *N* = 5, *P* < 0.0001) (see Supplementary Table 1 for details on statistical tests) and sMUA 1–5 Hz amplitude (*T*-TEST, ANESTHETIZED: *N* = 17, *P* < 0.0001, UNANESTHETIZED: *N* = 5, *P* < 0.0062) as illustrated in Figure 2E. When it is active, BL5 is also directly correlated with the CSD from L5 (median correlation over points with BL5 scaled amplitude > 2, *T*-TEST, ANESTHETIZED: *N* = 17, *P* < 0.0001, UNANESTHETIZED: *N* = 5, *P* < 0.0001) as well as the sMUA (*T*-TEST, ANESTHETIZED: *N* = 17, *P* < 0.0001, UNANESTHETIZED: *N* = 5, *P* = 0.0027) as shown in Figure 2F, where the correlation is taken over 6 s windows in the same manner as the amplitude. Both of these tests confirm that BL5 is correlated with local activity when BL5 is active.

We found that while THETA was correlated with the LFP (LFP scaled theta amplitude, *T*-TEST, ANESTHETIZED: *N* = 15, *P* = 0.0260, UNANESTHETIZED: *N* = 5, *P* = 0.0532; CSD L5, *T*-TEST, ANESTHETIZED: *N* = 17, *P* < 0.0001, UNANESTHETIZED: *N* = 5, *P* < 0.0001) as seen in Figure 2E, it was not correlated with the sMUA (sMUA scaled theta amplitude, *T*-TEST, ANESTHETIZED: *N* = 15, *P* = 0.4623, UNANESTHETIZED: *N* = 5, *P* = 0.5536; sMUA, *T*-TEST, ANESTHETIZED: *N* = 15, *P* = 0.4033, UNANESTHETIZED: *N* = 5, *P* = 0.1243) where results for individual experiments can be seen in Figure 2F. Since the THETA component was extracted from the LFP, we may expect that there is some correlation between them. However, the fact the THETA is not correlated with local spiking activity substantiates that THETA doesn’t have neocortical origin.

### THETA Amplitude Alternates With BL5 Amplitude

**FIGURE 3:**
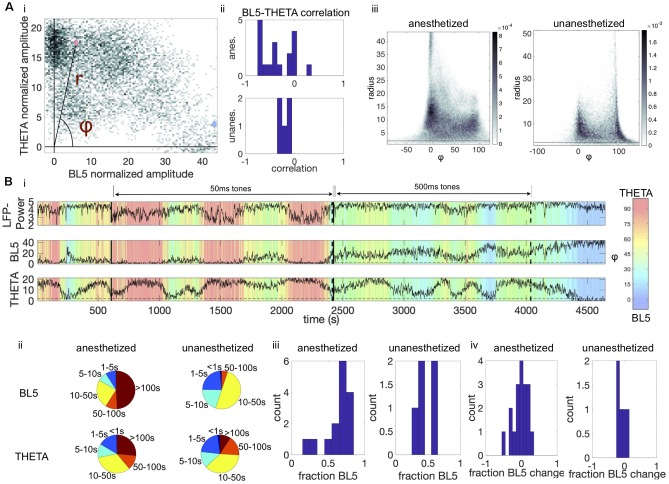
BL5 and THETA alternate within experiments. **(A,i)** Density plot of BL5 vs. THETA normalized amplitude for an example anesthetized experiment (same as Figure 1B). Pink and blue marks indicate points that are used for example BL5 and THETA active segments in Figure 1B, respectively. We set φ as the angle from the BL5-axis, while r is the radius. **(ii)** Correlations of BL5 vs. THETA normalized amplitude for each experiment. **(iii)** Conglomerate density plots of φ vs. radius for all anesthetized and unanesthetized experiments. **(B,i)** Time course of φ over the same example anesthetized experiment used in Figure 1. **(ii)** Pie charts showing the amount of time spent in BL5- vs. THETA-dominant episodes. For example, for anesthetized experiments during THETA-dominant states about 1/3 of time spent is in an episode of length 10–50 s. **(iii)** Histogram of the fraction of time spent in BL5- vs. THETA-dominant states. **(iv)** Histograms of the difference in fraction of time dedicated to BL5-dominant states during tone presentations vs. spontaneous activity.

Figure 2B appears to show that THETA and BL5 alternate with each other. To test this possibility, we calculated the correlation between the BL5 and THETA scaled amplitudes as illustrated in Figure 3Ai, and found that they were anticorrelated over all experiments (Figure 3Aii, *T*-TEST, ANESTHETIZED: *N* = 15, *P* = 0.0002, UNANESTHETIZED: *N* = 5, *P* = 0.0054) (see Supplementary Table 2 for details on statistical tests). Because BL5 and THETA are anti-correlated, we quantified the relative BL5-THETA participation in the CSD by calculating the angle φ and radius r for each BL5-THETA data point as shown in Figure 3Ai. In the conglomerate density plots of φ vs. r over all experiments (Figure 3Aiii), we can see peak densities where BL5 is dominant (φ ≤ 45) and where THETA is dominant (φ > 45). Thus, it makes sense to refer to BL5- and THETA-dominant states.

We then looked into how long BL5- and THETA-dominant states last. Figure 3Bi illustrates how φ changes over the entire example anesthetized experiment, where global trends tend to switch on the order of 100 s of seconds with finer-grained changes occurring throughout the experiment. Similar switching can be seen in unanesthetized experiments, as shown in Supplementary Figure 3. In Figure 3Bii, we see that although BL5-dominant states tend to last longer, both states can last on the order of 10–50 s. The amount of time spent in a BL5-dominant vs. THETA-dominant state was similar (Figure 3Biii, MEAN ± STANDARD DEVIATION (STD) ANESTHETIZED: 62.83 ± 16.56%, UNANESTHETIZED: 46.59 ± 8.71%). Furthermore, tones played during experiments do not appear to affect the length of states (Figure 3Biv, percent difference during tone vs. spontaneous activity, MEAN ± STD, ANESTHETIZED: 1.65 ± 18.39%, UNANESTHETIZED: -7.91 ± 14.72%).

### BL5 and THETA Amplitudes Alternate During Synchronized States

**FIGURE 4:**
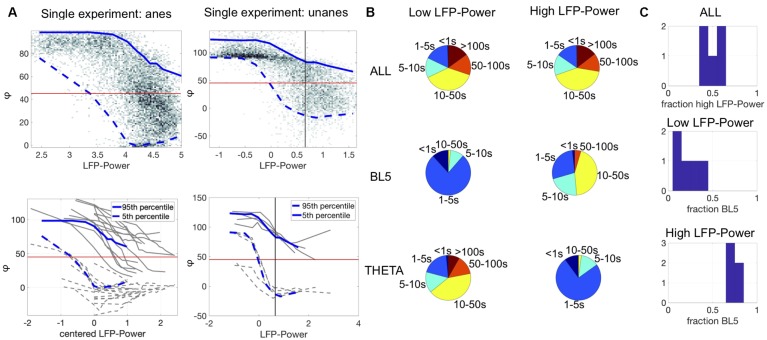
BL5 and THETA alternate within synchronized states. **(A)** Top plots show density of LFP-Power vs. φ for single experiments along with 5–95 percentile ranges. Bottom plots show ranges for all experiments with results for example experiments highlighted in blue. Anesthetized ranges are plotted against centered LFP-Power (LFP-Power minus LFP-Power with maximum range). Vertical black lines indicate median LFP-Power over unanesthetized experiments, used as the threshold between low vs. high LFP-Power. Note, by this measure all anesthetized experiments had high LFP-Power. **(B)** Distribution of episode lengths as a percentage of time spent in episodes of that length. **(C)** Fraction of time spent in each state. The top panel shows the fraction of time spent with high LFP-Power vs. low LFP-Power. The lower two panels show the fraction of time spent in BL5- vs. THETA-dominant states. Notably, approximately 30% of time in high LFP-Power is THETA-dominant. **(B,C)** Shows results for unanesthetized experiments only.

In Figure 2B, we see instances where either BL5 or THETA are active in the presence of noticeable CSD oscillations as seen during synchronized states. Synchronized states were previously characterized by high LFP-Power (Harris and Thiele, 2011). Since we showed in the above section that BL5 and THETA alternate within experiments, we further investigated whether BL5 and THETA alternate within synchronized states as well. Figure 4A shows the density plot of LFP-Power vs. φ for the same example experiments as Figure 2B and Supplementary Figure 2. Within this plot, φ ranges from 0 to 80 degrees for higher values of LFP-Power in these experiments. Calculating the range (5–95 percentile) over the density for all experiments reveals a wide range of φ where both BL5- and THETA-dominant states are included in the same echelons of LFP-Power. In contrast, φ often took high values (>80) when the LFP-Power is low.

We then explored how much time is spent in BL5- vs. THETA-dominant states when LFP-Power is high vs. when LFP-Power is low. Since many experiments didn’t have a clear threshold for LFP-Power, we set the threshold as the median LFP-Power over all unanesthetized experiments (Figure 4A and Supplementary Figure 4). With this threshold, virtually all anesthetized experiments had high LFP-Power, so we focused on unanesthetized experiments. The length of low vs. high LFP-Power episodes was fairly even, while THETA-dominant states were longer for low LFP-Power and BL5-dominant states were longer for high LFP-Power (see Figure 4B). When LFP-Power was low most of the time was spent in THETA-dominant states, while the amount of time spent in BL5- vs. THETA-dominant states when LFP-Power was high was similar to the ratio found in anesthetized experiments, with at least 25% of time spent in THETA-dominant states (Figure 4C, MEAN ± STD, PERCENT HIGH LPF-POWER: 50.33 ± 9.49%, BL5 AND LOW LFP-POWER: 19.06 ± 11.58%, BL5 AND HIGH LFP-POWER: 73.73 ± 3.34%). We further investigated the relationship between THETA amplitude and LFP-Power directly, since theta oscillations were previously noticed predominantly during desynchronized states, and not synchronized states (Diekelmann and Born, 2010). Supplementary Figure 5 reveals a similar relationship between LFP-Power and THETA oscillations as Figure 4A, with a wide range of amplitudes when LFP-Power is high. Furthermore, the length of episodes with significant THETA normalized amplitude during high LFP-Power is approximately the same as episodes when THETA is inactive (Supplementary Figure 5b). Likewise, about 50% of the time spent with high LFP-Power had significant THETA normalized amplitude (Supplementary Figure 5c, MEAN ± STD, PERCENT LOW SUB THETA, ANESTHETIZED: 37.16 ± 20.59%, UNANESTHETIZED: 34.86 ± 9.97%; LOW SUB THETA AND LOW LFP-POWER: 23.31 ± 11.66%, LOW SUB THETA AND HIGH LFP-POWER: 46.98 ± 6.53%).

**FIGURE 5:**
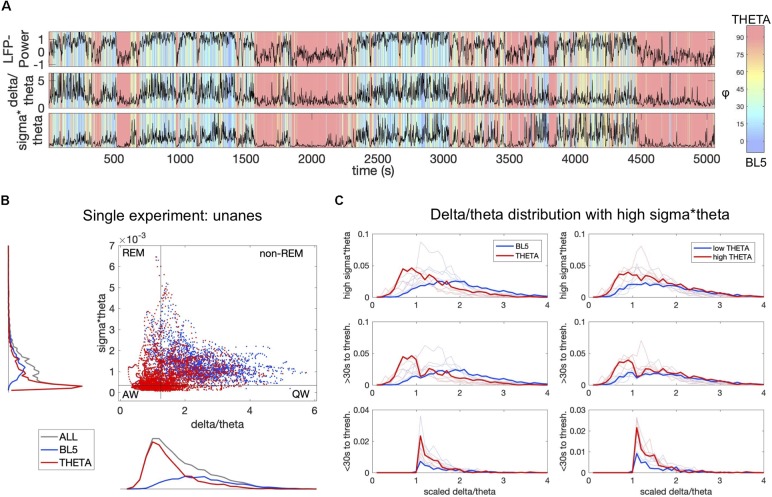
BL5 and THETA dominant states alternate within traditional sleep parameters. **(A)** LFP-Power, delta/theta power, and sigma × theta power compared to φ levels for the same unanesthetized experiment as Supplementary Figure 1. **(B)** THETA-dominant points (φ > 45) mapped on top of BL5-dominant points (φ ≤ 45) on the delta/theta vs. sigma × theta plane used to help distinguish sleep stages. The upper part of the plane indicates sleep, while the lower part indicates waking. High delta/theta indicates synchronized states such as non-REM sleep, while low delta/theta indicates desynchronized states such as REM sleep. Black boundaries indicate thresholds used for **(C)**. **(C)** Histograms of BL5- vs. THETA-dominant points on the left, and high THETA vs. low THETA amplitude on the right. Delta/theta is scaled by the delta/theta threshold for each experiment. Upper plots include all points above the sigma × theta threshold, set to be just above the peak sigma × theta density. Middle plots include points at least 30 s away from a transition across the delta/theta threshold. Lower plots include points within 30 s of a delta/theta threshold crossing. Results for example experiment highlighted in bold.

### BL5 and THETA Amplitudes Alternate Within Sleep Parameters

Since synchronized states may occur during both waking and sleeping, we further characterized the occurrence of BL5- vs. THETA-dominated states by comparing their amplitudes with traditional measures of sleep state. In particular, we calculated the delta (0.5–4 Hz), theta (6–9 Hz), and sigma (10–14 Hz) power for each unanesthetized experiment. We then compared φ with the measures sigma × theta, used to indicate wake vs. sleep, and delta/theta, used to indicate non-REM vs. REM sleep (Benington et al., 1994; Gross et al., 2009). Figure 5A shows how φ changes for a single unanesthetized experiment with respect to LFP-Power, delta/theta, and sigma × theta. Although the first few episodes with high LFP-Power and sigma × theta appear to be BL5-dominated, the last episode appears to have many points where THETA is dominant. We then compared BL5-dominated vs. THETA-dominated points directly on the delta/theta and sigma × theta plane. In Figure 5B, we can see that the top right quadrant which indicates non-REM sleep has clear overlap of both BL5 and THETA points. As expected, we predominantly see THETA-dominant states when delta/theta or sigma × theta is low. We divided the delta/theta and sigma × theta plane into regions (see section “Materials and Methods”) in order to estimate the percentage of time dedicated to BL5 vs. THETA. We found that, while episodes with BL5-dominant states tend to be longer within the estimated non-REM sleep region, a significant amount of time is spent in both BL5- and THETA-dominant states, with approximately 70 vs. 30% of time in each state (Supplementary Figure 6, percent BL5, MEAN ± STD: 5.05 ± 5.35% IN ACTIVE WAKE REGION, 9.76 ± 8.85% IN QUIET WAKE REGION, 30.62 ± 14.47% IN REM REGION, 68.46 ± 5.40% IN NON-REM REGION). Remarkably, the percentage of time spent with THETA-dominant is similar to high LFP-Power states. At the same time, approximately 50% of time within the estimated non-REM region has significant THETA amplitude (Supplementary Figure 6, MEAN ± STD PERCENT LOW SUB THETA: 12.10 ± 10.30% IN ACTIVE WAKE REGION, 18.32 ± 15.51% IN QUIET WAKE REGION, 26.23 ± 7.34% IN REM REGION, 46.51 ± 6.79% IN NON-REM REGION).

One reason for seeing theta during non-REM sleep could be the transition to REM state, where theta oscillations become strong just before REM (Benington et al., 1994; Gottesmann, 1996). To investigate whether THETA oscillations could represent the transition to REM, we plotted histograms of BL5- and THETA-dominant points over delta/theta values, where sigma × theta is above threshold shown in Figure 5C. We then removed points in the 30 s leading up to the delta/theta threshold, signifying a transition to REM. The histograms show that BL5- and THETA-dominated points have a similar distribution above the delta/theta threshold when transition points are removed. We furthermore checked the relationship of THETA amplitude with these sleep parameters directly. When we removed transitional points from the non-REM region, we also found similar distributions between high THETA amplitude vs. low THETA amplitude points. Together, these figures suggest that THETA-dominant states do not simply comprise transition-to-REM states.

### Neocortical Activity Differs Between BL5- and THETA-Dominant States

**FIGURE 6:**
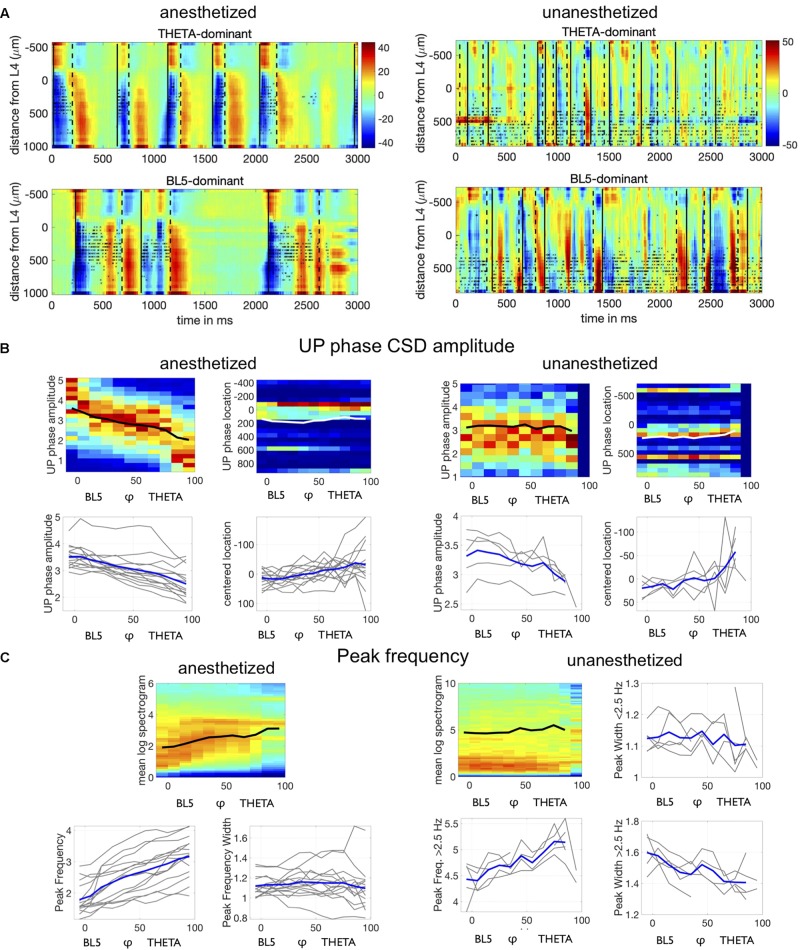
Neocortical activity differs depending on BL5- vs. THETA-dominant states. **(A)** CSD (color, mA/mm^3^) and spiking activity (black asterisks) from example anesthetized and unanesthetized experiments seen in previous figures. UP state onsets are marked by solid black lines, while DOWN state onsets are marked by dashed lines. **(B)** Top plot shows density of UP state amplitude and UP state amplitude peak location for individual experiments, along with the mean with respect to φ. Density is normalized for each φ bin, and the mean for a φ bin is calculated only if there are ≥ 30 points. We only included synchronized data points. Lower plots show the mean for all experiments, with highlighted results for the average over all experiment means. UP peak location is centered along the mean location for each experiment (UP location minus UP location mean). **(C)** Top left plot shows average spectrogram values over φ for the same example experiments as above, along with the mean peak frequency over φ. Other plots show means for all experiments, with highlighted results for the example experiment.

Figure 2B shows two versions of slow oscillatory activity in the L5 CSD and sMUA, one where BL5 is dominant and one where THETA is dominant. Looking at the CSD and spiking activity across neocortical layers in Figure 6A, we also notice qualitative differences in the activity between BL5- and THETA-dominant states. In particular, we noticed that BL5 states tended to have larger UP states centered in L5 with a slower frequency. At the same time, THETA states tended to have smaller UP states focused in L4 with a higher frequency.

To see if UP state CSD amplitude systematically changed with φ, we compiled the amplitude of all UP states during synchronized states (LFP-Power greater than median LFP-Power over all unanesthetized experiments). We noticed a down-ward trend in amplitude across φ in each experiment (see Figure 6B). We then tested whether UP state amplitude decreased with respect to φ independently from LFP-Power, because LFP-Power may also predict amplitude changes in individual UP states. We found that UP state CSD amplitude significantly decreased with φ beyond LFP-Power (TWO-WAY ANOVA WITH LFP-POWER AND φ  CONGLOMERATED OVER EXPERIMENTS, ANESTHETIZED: *P* < 0.0001, UNANESTHETIZED: *P* < 0.0001) (see Supplementary Table 3 for details on statistical tests). Since UP states appeared to be stronger in L5 during BL5-dominant states, we also tested the UP state amplitude peak neocortical depth. Similarly, we found that UP state neocortical depth decreased with respect to φ beyond LFP-Power (TWO-WAY ANOVA BETWEEN LFP-POWER ANDφ WITH EXPERIMENT AS RANDOM VARIATE, ANESTHETIZED: *P* < 0.0001, UNANESTHETIZED: *P* = 0.0023).

Finally, we investigated how the frequency content of the LFP changes with respect to φ. Figure 6C shows that the peak frequency tends to increase for anesthetized experiments, and the peak in the spectrogram narrows indicating that oscillations become more regular. We found that this trend is significant over all experiments independent of LFP-Power (two-way ANOVA between LFP-Power and φ, *p* = 0 for peak frequency, *p* = 0.008 for peak frequency width).

The frequency content of unanesthetized experiments proved to be richer than anesthetized experiments, with strong low frequency oscillations throughout synchronized states and an increasing envelope of delta frequencies. We tested whether the envelop of delta frequencies increased for all experiments by measuring the peak frequency above 2.5 Hz. We saw that the peak frequency increased significantly with φ beyond LFP-Power (TWO-WAY ANOVA WITH LFP-POWER AND φ, *P* < 0.0001). The peak frequency below 2.5 Hz did not change significantly with φ. However, the peak frequency width appeared to narrow with φ, indicating that oscillations became more regular. We saw that the peak frequency width decreased significantly over all unanesthetized experiments both below 2.5 Hz (TWO-WAY ANOVA WITH LFP-POWER AND φ, *P* < 0.0001) and above 2.5 Hz (TWO-WAY ANOVA WITH LFP-POWER AND φ, *P* < 0.0001), indicating that both facets of the frequency spectra became more regular.

## Discussion

We applied ICA to recordings from rat AC and consistently found two components that alternate within synchronized states. The BL5 component has a high amplitude and follows the overall activity. The THETA component generates a theta oscillation which appears to orginate below the neocortex. Neocortical UP state activity varies depending on which component is active. These results indicate that there are two modes of operation within synchronized states.

### Related Studies on Differences Within Synchronized States and Application of ICA

There are several other studies that note different modes within synchronized states. Recently, Miyawaki et al. (2017) describe low-amplitude synchronized states (LOW) seen in the hippocampus, which builds on previously noted small-irregular activity. LOW states may be related to our THETA-dominant states since UP state CSD amplitude is lower during THETA-dominant states. Notably, LOW states occur simultaneously in the hippocampus, entorhinal cortex, neocortex, and thalamus. Therefore, if there is a correlation between LOW states and THETA-dominant states, then low-amplitude irregular activity should be seen in the hippocampus during synchronized THETA-dominant states. Thus, our study hints that there are underlying theta oscillations during low-amplitude states in the hippocampus.

Although rat hippocampal theta oscillations have only been noted during desynchronized states in most studies, there are a few studies that look at theta oscillations during synchronized states. Hippocampal theta is seen in non-REM sleep during transition-to-REM states shortly before REM starts (Benington et al., 1994; Robert et al., 1999). However, transition-to-REM lasts up to 10 s (Gottesmann, 1996), whereas THETA-dominant states can last significantly longer and do not necessarily lead to REM sleep. Several studies have noted that theta power in the hippocampus and thalamus during non-REM sleep can be as strong as theta power during REM sleep (Gaztelu et al., 1994; Pedemonte et al., 2005), but these oscillations may be more difficult to observe because of ongoing irregular activity. Finally, theta oscillations can be seen with increased tiredness during waking states (Vyazovskiy and Tobler, 2005).

Independent component analysis is a form of analysis that separates overlapping signals in a set of recordings (Bell and Sejnowski, 1995; Amari et al., 1996; Hyvärinen and Oja, 1997; Hyvärinen and Oja, 2000; Brown et al., 2001; Herreras et al., 2015). Previously, ICA has been used a great deal in artifact removal as well as identifying regions of interest in fMRI and EEG (McKeown et al., 2003; Onton et al., 2006; Eichele et al., 2009; Esposito and Goebel, 2011; Huster et al., 2015; Calhoun and de Lacy, 2017). More recently, ICA was used in LFP recordings to parse population involvement in the hippocampus (Makarov et al., 2010; Agarwal et al., 2014; Herreras et al., 2015; Benito et al., 2016). To our knowledge, this is the first study to use ICA to study synchronized states.

### Interpretation of Results With ICA and Limitations

One reason why ICA has not been used more on LFP recordings to date may be because LFP recordings have access to relatively detailed information, so there are more options available for analysis than fMRI and EEG. At the same time, ICA is limited in the fact that the number of components it can return is limited by the number of recordings. Because there are potentially more populations detected by neural recordings than can possibly be parsed, it has been unclear how ICA would behave when faced with real neural data. Recent simulations in Makarov et al. (2010) and Głabska et al. (2014) show that ICA tends to group similar populations together, which is consistent with our results. With more recordings, ICA would be able to pick out more sources at finer resolution (Agarwal et al., 2014; Jun et al., 2017).

As ICA groups populations with similar anatomical profiles together, BL5 and THETA may not stand for single neural populations. In fact, ICA will parse any signal that affects recordings, including glia and movement artifacts. ICA components also may be composed of sets of synapses onto cells acting synchronously (Herreras et al., 2015). Furthermore, it is possible that two populations that act independently at some time points, and synchronously at others, may be separated into three components: one component for each of the populations individually and a third for the combination (Herreras et al., 2015).

Although we consistently found a THETA component which likely exhibited sub-neocortical theta oscillations, the theta oscillations were more difficult to see in unanesthetized experiments compared to anesthetized experiments during synchronized states. In particular, the theta oscillation was often mixed with other low-frequency activity in unanesthetized experiments, while the theta peak was isolated in all anesthetized experiments. The theta-frequency peak may have been clearer for anesthetized THETA components because: (1) activity under anesthesia is simpler than activity without anesthesia and so ICA could better isolate the theta oscillations in anesthetized experiments, or (2) the THETA ICA component in unanesthetized experiments included movement artifact which allowed neocortical signal to leak into the component. In the first case, the THETA component may even isolate theta oscillations from multiple sources that originate below the cortex. For instance, the hippocampus, which is directly below the AC and is known to show mixed frequencies during synchronized states (Buzsáki, 2002).

Another limitation of our study is that instead of sleep staging, we compared ICA components to sleep stage parameters. Thresholds for these parameters are based on the data from rat recordings lasting 1–1.5 h, which may not include all sleep stages. This means that sleep regions may not necessarily line up with sleep stages seen in other experiments. While our analysis does not provide a direct comparison with sleep stages, a comparison to the parameters allows us to see how BL5 and THETA signals relate to each other regardless of how sleep stages may be decided. Likewise, while there is no exact threshold for LFP-Power to determine synchronized vs. desynchronized states, we are still able to compare LFP-Power with BL5 and THETA signals.

### Possible Implications for Synchronized States and Further Research

Our results show that BL5 UP states have relatively high amplitude, while THETA UP states have lower amplitude. Amplitude during sleep has previously been characterized by SWA, which is high at sleep onset and slowly subsides throughout the night (Dijk et al., 1990; Aeschbach and Borbély, 1993; Vassalli and Dijk, 2009; Rodriguez et al., 2016). Because of this change, we hypothesize that BL5-dominant states may be emphasized during early sleep, while THETA-dominant states may be emphasized later, consistent with LOW states (Miyawaki et al., 2017). According to the two-process model of sleep, early sleep is associated with homeostatic processes along with SWA (Borbély, 1982; Achermann et al., 1993; Borbély et al., 2016). At the same time, sleep is hypothesized to serve two functions: synaptic homeostasis and memory consolidation (Vassalli and Dijk, 2009; Diekelmann and Born, 2010; Rasch and Born, 2013). In particular, late sleep and lower amplitude stage 2 sleep has been shown to benefit certain types of memory consolidation (Diekelmann and Born, 2010; Rasch and Born, 2013; Hutchison and Rathore, 2015).

If BL5 and THETA reflect different modes within synchronized states, then what populations could they represent? BL5 encompasses much of the cortex, is relatively high amplitude, and mimics the CSD. This means that BL5 could comprise a large population within the cortex, a high amplitude population, or both. Early work shows that glial recordings have relatively high amplitude and follow the LFP (Amzica and Steriade, 1998). Glia are also known to be involved in homeostatic processes during sleep (Clinton et al., 2011; de Andrés et al., 2011; Poskanzer and Yuste, 2011; Fellin et al., 2012; Bjorness et al., 2016). Furthermore, recent work with calcium imaging shows heavy glial involvement prior to neuronal involvement during SWA (Szabó et al., 2017). Therefore, BL5 may include glia.

THETA represents a population exhibiting sub-neocortical theta oscillations. Theta oscillations are clock-like sinusoidal oscillations previously reported in the hippocampus along with other limbic structures (Buzsáki, 2002). Since the hippocampus lies directly below the rat auditory neocortex, then THETA may originate from the hippocampus or possibly deeper limbic structures (Sirota et al., 2008; Kajikawa and Schroeder, 2011). At the same time, the theta oscillations we observed had a lower frequency, matching type 2 theta oscillations previously associated with sensory processing and emotionally salient stimuli during waking states (Bland and Oddie, 2001; Tendler and Wagner, 2015). If type 2 theta oscillations are associated with emotionally relevant memory processing, then they could also be linked to hippocampal-amygdala co-activation (Pelletier and Paré, 2004; Hutchison and Rathore, 2015). It has also been hypothesized that theta oscillations correspond to information coming in to the hippocampus, while sharp waves indicate information transferred out of the hippocampus (Hasselmo, 1999). THETA oscillations during synchronized states may mean that there are low-amplitude theta oscillations within the hippocampus at the same time as sharp waves, where the theta oscillations stand for information coming in from another structure such as the amygdala, while information is begin transferred out via sharp waves.

Further research would involve verification of the roles of various populations in BL5- and THETA-dominant states. Being able to focus on one state within synchronized states may also help to shed light on not only mechanisms for oscillatory activity, but functional roles for activity. For instance, memory processing has been shown to be hampered from sleep deprivation for the latter half of the night (Rasch and Born, 2013). Previously, this was tied to a lack of REM sleep which plays a larger role later in the night. However, stage 2 non-REM sleep frequently alternates with REM in the latter half of the night and further study may elucidate a unique role for this stage in memory processing (Diekelmann and Born, 2010; Rasch and Born, 2013; Hutchison and Rathore, 2015).

We may also use this analysis to find different modes of operation, and specifically theta oscillations, in other species. For example, while rat hippocampal theta is easy to pick out, theta oscillations tend to be more difficult to identify and study in other species, such as humans and bats (Jacobs, 2013).

## Conclusion

Independent component analysis reveals that theta oscillations exist within synchronized states, and that they alternate with a high-amplitude population in the neocortex. These populations are associated with different neocortical activity. Therefore, these data indicate that synchronized states are not uniform, but may be separated according to at least two distinct functional roles.

## Data Availability

The datasets generated for this study can be access at https://doi.org/10.15129/1225d3ad-9728-43a9-8091-5259f091f7a7 or are available on request to the corresponding author.

## Author Contributions

EMK: conceptualization, formal analysis, and writing – original draft. EMK and TT: methodology. SS: *in vivo* experiments. TT and SS: resources. EMK, TT, and SS: writing – review and editing. TT: supervision.

## Conflict of Interest Statement

The authors declare that the research was conducted in the absence of any commercial or financial relationships that could be construed as a potential conflict of interest.
