# Corrigendum: Theta Oscillations Alternate With High Amplitude Neocortical Population Within Synchronized States

**DOI:** 10.3389/fnins.2019.00631

**Published:** 2019-06-25

**Authors:** Erin Munro Krull, Shuzo Sakata, Taro Toyoizumi

**Affiliations:** ^1^RIKEN Center for Brain Science, Tokyo, Japan; ^2^Beloit College, Beloit, WI, United States; ^3^Strathclyde Institute of Pharmacy and Biomedical Sciences, University of Strathclyde, Glasgow, United Kingdom

**Keywords:** synchronized state, independent component analysis, theta oscillation, non-REM, slow oscillation, delta oscillation, cortical state

In the original article, there was a mistake in the legend for Figure 6 as published. In panels B and C, the average over all means is highlighted instead of the means for example experiments in panel A. The correct legend appears below.

**Figure 6** | Neocortical activity differs depending on BL5- vs. THETA-dominant states. **(A)** CSD (color, mA/mm^3^) and spiking activity (black asterisks) from example anesthetized and unanesthetized experiments seen in previous figures. UP state onsets are marked by solid black lines, while DOWN state onsets are marked by dashed lines. **(B)** Top plot shows density of UP state amplitude and UP state amplitude peak location for individual experiments, along with the mean with respect to *φ*. Density is normalized for each *φ* bin, and the mean for a *φ* bin is calculated only if there are ≥ 30 points. We only included synchronized data points. Lower plots show the mean for all experiments, with highlighted results for the average over all experiment means. UP peak location is centered along the mean location for each experiment (UP location minus UP location mean). **(C)** Top left plot shows average spectrogram values over *φ* for the same example experiments as above, along with the mean peak frequency over *φ*. Other plots show means for all experiments, with highlighted results for the example experiment.

In the original article, there was also a mistake in Supplementary Table 3 as published. There is an error in UP Depth Anes. SUB Mean, Peak Frequency (<2.5 Hz) Unanes. BL5 Mean, and Peak Frequency Width (>2.5 Hz) Unanes. Test Statistic. The corrected Supplementary Table 3 appears below.

**Supplementary Table 3:** Statistical tests accompanying results in Figure 5.

**CONDITION**	**N**	**P-VALUE**	**TEST STATISTIC**	**EFFECT SIZE**	**BL5 MEAN**	**BL5 SEM**	**SUB MEAN**	**SUB SEM**
**UP NORMALIZED AMPLITUDE, ANES**	115444	0	6.5187E3	5.3244E-2	3.3595	0.0046	2.7518	0.0059
**UP NORMALIZED AMPLITUDE, UNANES**	21959	1.2692E-13	54.9684	2.4972E-3	3.2779	0.0106	3.1046	0.0059
**UP DEPTH, ANES**	115444	6.2071E-18	74.4782	5.5583E-4	239.8261	1.2229	220.6725	1.719
**UP DEPTH, UNANES**	21959	0.0023	9.2942	3.8785E-4	110.228	2.7267	93.4306	4.9412
**PEAK FREQUENCY, ANES**	98243	0	7.5526E3	6.4653E-2	2.1705	0.0039	2.7343	0.0051
**PEAK FREQUENCY** ** <2.5 HZ, UNANES**	16551	0.6483	0.2081	1.2457E-5	1.3549	0.0054	1.3600	0.0096
**PEAK FREQUENCY** **>2.5 HZ, UNANES**	16551	6.4779E-8	29.2416	1.6102E-3	4.5372	0.0163	4.7166	0.0287
**PEAK FREQUENCY WIDTH, ANES**	98243	8.0711E-4	11.2255	1.1415E-4	1.1264	0.0022	1.1140	0.0029
**PEAK FREQUENCY WIDTH** ** <2.5 HZ, UNANES**	16551	7.3699E-7	24.5348	1.4778E-3	1.1529	0.0044	1.1084	0.0078
**PEAK FREQUENCY WIDTH** **>2.5 HZ, UNANES**	16551	3.7843E-12	48.3050	2.8986E-3	1.5664	0.0071	1.4661	0.0125

*Matlab anovan() used throughout. Means and standard error of the mean (SEM) calculated with multcompare(). Effect size is the sum of squares divided by total sum of squares*.

The authors apologize for this error and state that this does not change the scientific conclusions of the article in any way. The original article has been updated.

