# Supplementary material for: Theta Oscillations Alternate With High Amplitude Neocortical Population Within Synchronized States
Source: Front Neurosci. 2019 Apr 12;13:316. doi: 10.3389/fnins.2019.00316 (PMC6476345; doi:10.3389/fnins.2019.00316)
Supplement: Supplementary file 1 [file Data_Sheet_1.docx]

Supplementary Material

# Supplementary Figures and Tables

## Supplementary Figures


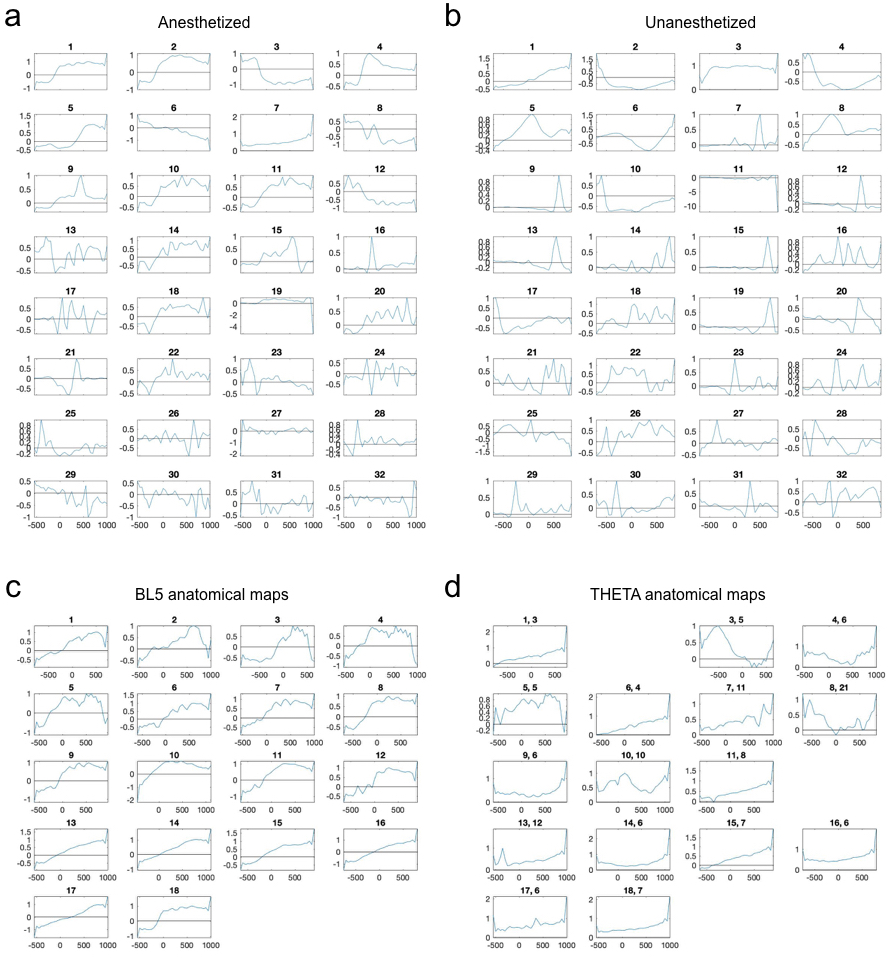


**Supplementary Figure 1.** *Anatomical maps. (a) All anatomical maps for the example anesthetized experiment. ICA component number associated with the map is displayed above. ICA sorts all components from highest amplitude to lowest amplitude. In this experiment, ICA component 1 is BL5, while ICA component 7 is THETA. (b) All anatomical maps of the example unanesthetized experiment. ICA component 1 is BL5, while ICA component 3 is THETA. Note that THETA is the only component with no dipole. (c) BL5 anatomical maps for anesthetized experiments. (d) THETA anatomical maps for anesthetized experiments. Experiment number along with component number is shown above each map. Note that experiments 2 and 12 did not exhibit a THETA component.*


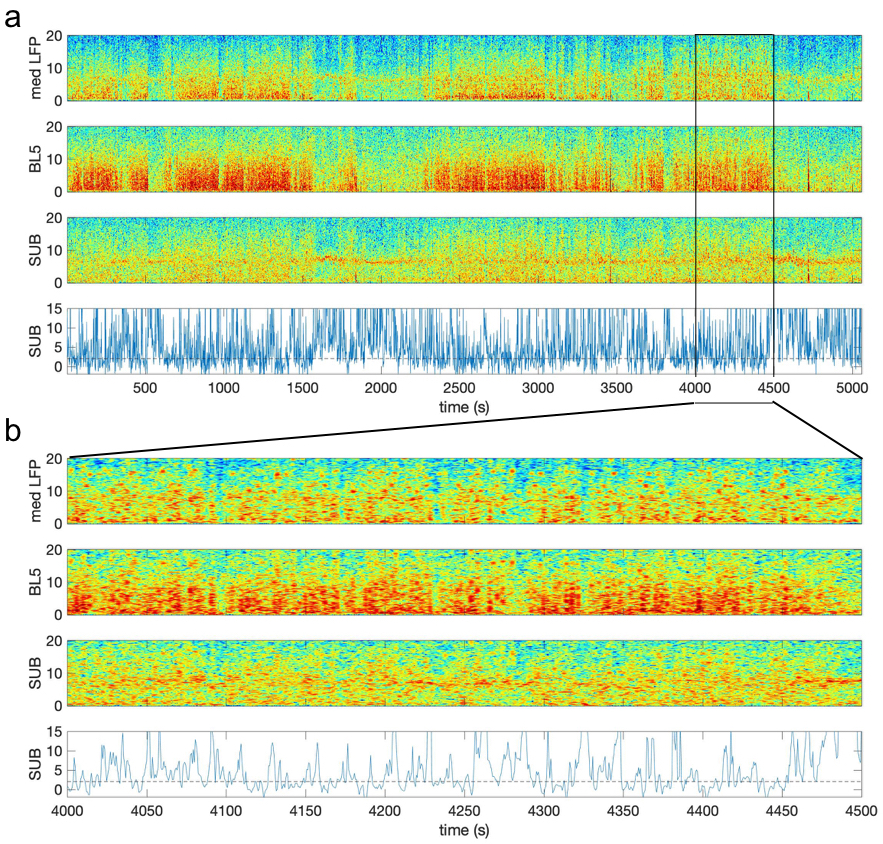


**Supplementary Figure 2.** Spectrograms of an example unanesthetized experiment along with SUB scaled amplitude. (a) The top graph shows the median spectrogram of the LFP recorded over all channels. LFP-Power is the summed power from 1-5 Hz of this spectrogram. The BL5 spectrogram shows intermittent strong low frequency oscillations. The SUB spectrogram clearly exhibits a theta oscillation when LFP-Power is low. (b)Close-up of the spectrograms from panel a when LFP-Power is high. The SUB spectrogram exhibits intermittent theta, while the surrounding frequencies may be higher than when LFP-Power is low. In order to not over-estimate the SUB scaled amplitude when LFP-Power is high, we scaled the amplitude with respect to surrounding frequencies within a 6 s window instead of the entire experiment. As a result, SUB amplitude is compared to the relatively higher amplitude of local surrounding frequencies. Despite this, we see theta amplitudes that are larger than surrounding frequencies according to the SUB scaled amplitude shown in the bottom panel. The SUB scaled amplitude is considered significant when it is above 2, noted by a black dashed line.

*
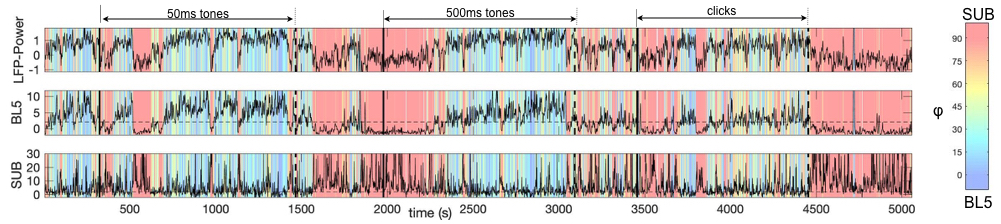
*

**Supplementary Figure 3**. BL5 and SUB alternate throughout an unanesthetized experiment used as an example in this study. Colors reflect phi values, which represent relative BL5 ($\varphi$ ≤45) vs. SUB ($\varphi$ >45) involvement. This experiment features four segments where LFP-Power is high. The first three segments appear to contain mostly BL5, while the last segment is largely SUB-dominant. Within these regions, we also see finer grained switching between BL5 and SUB.


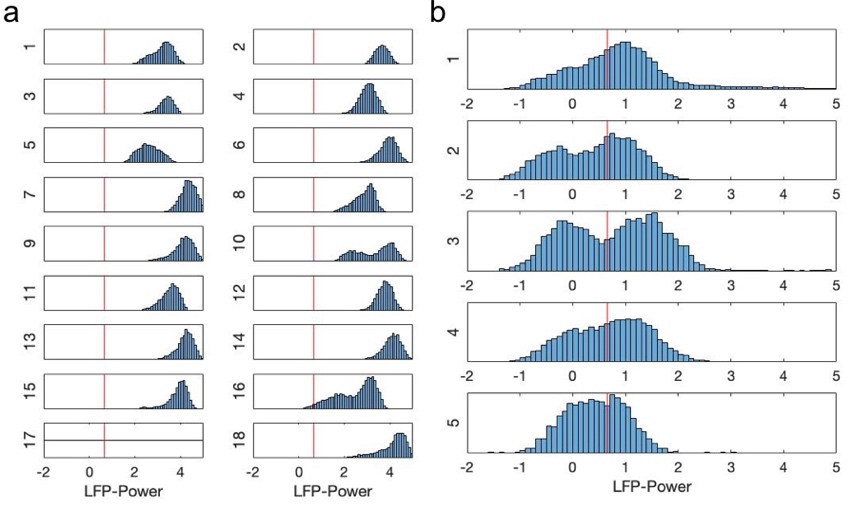


**Supplementary Figure 4**. Distribution of LFP-Power over individual experiments, along with median LFP-Power over all unanesthetized experiments. (a) Anesthetized experiments. (b) Unanesthetized experiments.


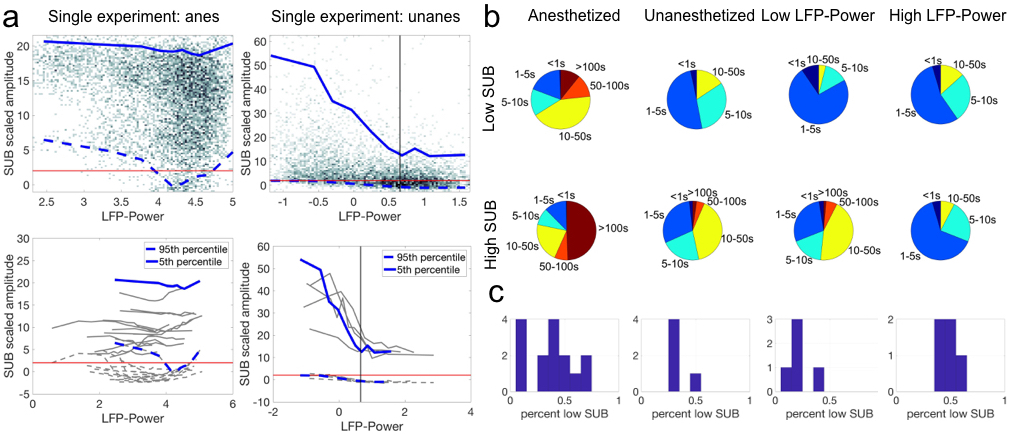


**Supplementary Figure 5.** Relationship between SUB scaled amplitude and LFP-Power. (a) Similarly to $\varphi$, there is a wide range of SUB scaled amplitude over high values of LFP-Power. Top plots show density of LFP-Power vs. SUB scaled amplitude along with 5-95 percentile range outlined in blue. Bottom plots show the range calculated for all experiments, with example experiments highlighted in blue. Black vertical lines indicate threshold for low vs. high LFP-Power used to calculate state durations and time percentages. Red horizontal lines indicate threshold SUB scaled amplitude of 2. (b) State durations of low vs. high LFP-Power and low vs. high SUB scaled amplitude. (c) Percentage of time spent with low SUB scaled amplitude. Note that SUB scaled amplitude is significant approximately 50% of time during high LFP-Power.


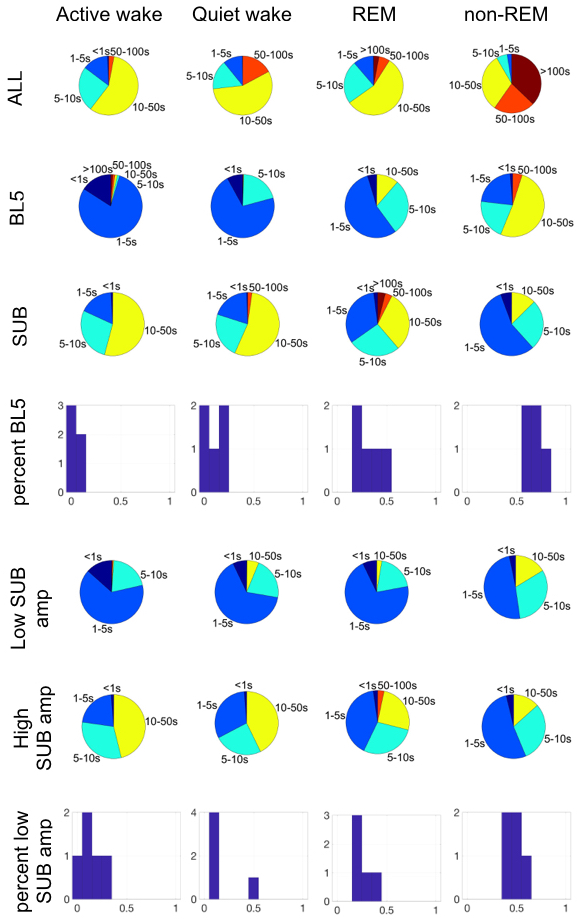


**Supplementary Figure 6**. Time durations with respect to sleep parameters. Note that approximately 70% of time in non-REM regions is BL5-dominant, while 30% is SUB-dominant, similarly to states with high LFP-Power. Likewise, approximately 50% of time in non-REM regions has significant SUB scaled amplitude.

| condition | test | n | p-value | df | test statistic | mean | std |
| --- | --- | --- | --- | --- | --- | --- | --- |
| correltion between LFP-Power and BL5 scaled amp, anes | t-test | 17 | 7.4863E-07 | 16 | 7.8163 | 0.5478 | 0.2890 |
| correltion between LFP-Power and BL5 scaled amp, unanes | t-test | 5 | 2.2632E-04 | 4 | 12.6679 | 0.7659 | 0.1352 |
| correltion between sMUA LFA and BL5 scaled amp, anes | t-test | 17 | 1.1480E-05 | 16 | 6.2555 | 0.4584 | 0.3022 |
| correltion between sMUA LFA and BL5 scaled amp, unanes | t-test | 5 | 0.0062 | 4 | 5.2803 | 0.4686 | 0.1984 |
| correltion between LFP relative theta and SUB scaled amp, anes | t-test | 15 | 0.0260 | 14 | 2.4884 | 0.1398 | 0.2175 |
| correltion between LFP relative theta and SUB scaled amp, unanes | t-test | 5 | 0.0532 | 4 | 2.7161 | 0.1298 | 0.1069 |
| correltion between sMUA relative theta and SUB scaled amp, anes | t-test | 15 | 0.4623 | 14 | 0.7558 | 0.0286 | 0.1465 |
| correltion between sMUA relative theta and SUB scaled amp, unanes | t-test | 5 | 0.5536 | 4 | 0.6459 | 0.0064 | 0.0223 |
| median correlation between CSD L5 and BL5 when active, anes | t-test | 17 | 3.5977E-12 | 16 | 18.3486 | 0.6233 | 0.1401 |
| median correlation between CSD L5 and BL5 when active, unanes | t-test | 5 | 1.3056E-04 | 4 | 14.5274 | 0.6595 | 0.1015 |
| median correlation between sMUA and BL5 when active, anes | t-test | 17 | 1.8073E-10 | 16 | -14.1601 | -.3716 | 0.1082 |
| median correlation between sMUA and BL5 when active, unanes | t-test | 5 | 0.0027 | 4 | -6.5997 | -.2018 | 0.0684 |
| median correlation between CSD L5 and SUB when active, anes | t-test | 15 | 9.7414E-05 | 14 | 5.3779 | 0.1468 | 0.1057 |
| median correlation between CSD L5 and SUB when active, unanes | t-test | 5 | 1.3252E-04 | 4 | 14.4726 | 0.4246 | 0.0656 |
| median correlation between sMUA and SUB when active, anes | t-test | 15 | 0.4033 | 14 | -.8619 | -.0154 | 0.0690 |
| median correlation between sMUA and SUB when active, unanes | t-test | 5 | 0.1243 | 4 | -1.9408 | -.0353 | 0.0406 |

**Supplementary Table 1**. Statistical tests accompanying histograms in Fig. 1de. Matlab ttest() used throughout. DF=degrees of freedom. STD=standard deviation.

| condition | test | n | p-value | df | test statistic | mean | std |
| --- | --- | --- | --- | --- | --- | --- | --- |
| BL5-SUB scaled amplitude correlation, anes | t-test | 15 | 1.6812E-04 | 14 | -5.0788 | -.4027 | 0.3071 |
| BL5-SUB scaled amplitude correlation, unanes | t-test | 5 | 0.0054 | 4 | -5.4749 | -.2105 | 0.0860 |

**Supplementary Table 2**. Statistical tests accompanying histograms in Fig. 2aii. Matlab ttest() used throughout. DF=degrees of freedom. STD=standard deviation.

| condition | n | p-value | test statistic | effect size | BL5 mean | BL5 SEM | SUB mean | SUB SEM |
| --- | --- | --- | --- | --- | --- | --- | --- | --- |
| UP normalized amplitude, anes | 115444 | 0 | 6.5187E3 | 5.3244E-2 | 3.3595 | 0.0046 | 2.7518 | 0.0059 |
| UP normalized amplitude, unanes | 21959 | 1.2692E-13 | 54.9684 | 2.4972E-3 | 3.2779 | 0.0106 | 3.1046 | 0.0059 |
| UP depth, anes | 115444 | 6.2071E-18 | 74.4782 | 5.5583E-4 | 239.8261 | 1.2229 | 220.6725 | 1.719 |
| UP depth, unanes | 21959 | 0.0023 | 9.2942 | 3.8785E-4 | 110.228 | 2.7267 | 93.4306 | 4.9412 |
| Peak frequency, anes | 98243 | 0 | 7.5526E3 | 6.4653E-2 | 2.1705 | 0.0039 | 2.7343 | 0.0051 |
| peak frequency <2.5 Hz, unanes | 16551 | 0.6483 | 0.2081 | 1.2457E-5 | 1.3549 | 0.0054 | 1.3600 | 0.0096 |
| peak freqeuncy >2.5 Hz, unanes | 16551 | 6.4779E-8 | 29.2416 | 1.6102E-3 | 4.5372 | 0.0163 | 4.7166 | 0.0287 |
| peak frequency width, anes | 98243 | 8.0711E-4 | 11.2255 | 1.1415E-4 | 1.1264 | 0.0022 | 1.1140 | 0.0029 |
| peak frequency width <2.5 Hz, unanes | 16551 | 7.3699E-7 | 24.5348 | 1.4778E-3 | 1.1529 | 0.0044 | 1.1084 | 0.0078 |
| peak frequency width >2.5 Hz, unanes | 16551 | 3.7843E-12 | 48.3050 | 2.8986E-3 | 1.5664 | 0.0071 | 1.4661 | 0.0125 |

**Supplementary Table 3**. Statistical tests accompanying results in Fig. 5. Matlab anovan() used throughout. Means and standard error of the mean (SEM) calculated with multcompare(). Effect size is the sum of squares divided by total sum of squares.
